# Longitudinal Relations Among Theory of Mind, Advanced Theory of Mind, and Executive Function From Ages Four to Seven

**DOI:** 10.1111/desc.70173

**Published:** 2026-03-19

**Authors:** Christopher Osterhaus, Beate Sodian, Özgün Köksal, David M. Sobel

**Affiliations:** ^1^ Developmental Psychology in Education University of Vechta Vechta Germany; ^2^ Department of Psychology Ludwig‐Maximilians‐Universität München Munich Germany; ^3^ Department of Cognitive and Psychological Science Brown University Providence Rhode Island USA

**Keywords:** advanced theory of mind (AToM), conceptual development, executive function (EF), theory of mind (ToM)

## Abstract

This longitudinal study examined the developmental relations between first‐order Theory of Mind (ToM), advanced ToM, and executive function (EF) from ages 4 to 7.5. Two‐hundred‐three German children were assessed at ages 4, 5.5, and 7.5 on measures of ToM, EF (working memory, inhibition, cognitive flexibility), general cognition, and language. Using regression, structural equation modeling (SEM), and latent class analysis, we investigated whether early ToM and EF predict advanced ToM performance and how the developmental pathways unfold. We found significant concurrent relations between ToM and EF at all time points, as well as a predictive relation from EF at age 4 to advanced ToM at age 7.5. ToM at age 4 also significantly predicted ToM at age 7.5, suggesting early ToM lays a foundation for later conceptual growth. Cross‐lagged SEM revealed that EF at age 4—but not at 5.5—predicted advanced ToM, indicating EF may support ToM development at key transition points. Latent class analysis identified four ToM profiles: consistently high performers, late bloomers, partial achievers, and inconsistent performers. Early language skills and later inhibition differentiated these groups; general cognition played a lesser role. These findings support a hybrid account of ToM development: early conceptual understanding is foundational for later competence, but qualitative shifts are required to master more complex ToM forms. EF and language appear as critical supports for ToM development, especially during periods of conceptual change. This study contributes to a more nuanced view of how domain‐general and domain‐specific processes interact in the development of complex social cognition.

**Summary:**

- Longitudinal study of Theory of Mind (ToM), advanced ToM, and executive function (EF) from ages 4 to 7.5.
- ToM and early EF at age 4 significantly predicted advanced ToM performance at age 7.5, independent of general cognitive ability.
- Latent class analysis identified four distinct developmental ToM pathways, including partial achievers and inconsistent performers.
- Findings support a hybrid view: early conceptual continuity lays a foundation, but later conceptual change is needed for advanced mental state reasoning.

## 1 Introduction

Theory of Mind (ToM) refers to the understanding that human behavior and social interactions are driven by agents’ unobservable, internal mental states (Astington 1993; Gopnik and Astington 1988; Perner et al. 1987; Premack and Woodruff 1978; Wellman 2014). The study of ToM spans lifelong development, including infancy (e.g., Onishi and Baillargeon 2005; Scott and Baillargeon 2017; Sodian 2011; Wang et al. 2016), adolescence (e.g., Bosacki 2021; Dumontheil et al. 2012), and adulthood (e.g., Apperly et al. 2009). Most research on ToM, however, centers on preschoolers, particularly the acquisition of the representational capacities necessary to succeed on measures of false belief (Perner 1991; Sobel 2023). A large body of evidence indicates that understanding that others can represent the world differently from the actual state of the world develops between the ages of 3 and 5 and is a milestone in ToM development (Wellman et al. 2001).

Recent research, however, has challenged this view by indicating that even toddlers, before their third birthday, succeed in understanding others’ false beliefs when processing demands are appropriately lowered (Setoh et al. 2016), and that infants are sensitive to false belief information in spontaneous response tasks (Baillargeon et al. 2010; but see reports on failed replications, Poulin‐Dubois et al. 2018). These findings support the view that there may be high‐level conceptual continuity, rather than fundamental conceptual change in children's acquisition of a basic understanding of the mental domain (Scott et al. 2017; Sodian et al. 2020).

This theoretical perspective may also be influential on how ToM develops during the elementary school years. A growing literature highlights significant developmental progressions in ToM during the elementary school years (e.g., Banerjee et al. 2011; Devine and Hughes 2016; Lecce et al. 2017; Osterhaus and Koerber 2021a; Peterson et al. 2012): Elementary‐school children develop an understanding of increasingly complex forms of higher‐order false belief, encompassing second‐ and third‐order false belief understanding (e.g., Liddle and Nettle 2006; Perner and Wimmer 1985; Sullivan et al. 1994). Additionally, they acquire the ability to comprehend nonliteral speech, such as irony or jokes (Happé 1994; White et al. 2009), interpret emotions and mental states from facial expressions (Baron‐Cohen et al. 2001), identify breaches of social norms, such as faux pas (Baron‐Cohen et al. 1999), and recognize individual differences in interpreting stimuli based on knowledge and perspective (Carpendale and Chandler 1996; Osterhaus and Bosacki 2022). Collectively, these abilities, often grouped under the umbrella term *advanced theory of mind* (advanced ToM), undergo significant development between ages 5–10 (Osterhaus et al. 2016; Osterhaus and Koerber 2021a).

### 1.1 From First‐Order to Advanced Theory of Mind: Conceptual Continuity or Conceptual Change?

First‐order and advanced ToM abilities have been well‐characterized independently; the developmental and conceptual continuity between these levels of ToM—particularly the transition from early false‐belief understanding to higher‐order mental state reasoning—remains insufficiently understood. This gap in the literature raises important questions about the nature of ToM development: Does progression from first‐order to advanced ToM reflect a qualitative shift in children's conceptual understanding of mental states, or is it primarily driven by an underlying conceptual continuity with change the result of general cognitive growth, such as improved executive functioning (EF) and processing capacities?

Two main hypotheses have been proposed to explain the transition from first‐order to advanced ToM. According to the *Conceptual‐Development Hypothesis* (Perner 1988), advanced ToM requires qualitatively new conceptual insights. Specifically, children must progress from understanding that others can hold false beliefs (first‐order) to grasping the recursive nature of mental states—that is, that one person can hold a belief about another's belief (second‐order). In contrast, the *Complexity‐Only Hypothesis* (Sullivan et al. 1994) posits that advanced ToM tasks do not require additional conceptual insights but rather advanced processing skills such as increased EF capacities. On this view, advanced ToM tasks involve the same basic concepts as first‐order ToM but place greater demands on domain‐general processing resources, such as language, working memory, and inhibitory control.

These two accounts make different predictions about the role of EF in ToM development. If advanced ToM reflects increasing processing demands, then individual differences in EF should be a consistent, longitudinal predictor of ToM across development, and especially of later performance on advanced tasks. In contrast, if advanced ToM requires new conceptual insights, EF should be most predictive at specific transition points, when children must restructure how they represent beliefs and belief recursion, rather than exerting a uniform influence across time.

Evidence exists for both perspectives. Supporting the complexity‐only hypothesis, reducing the complexity of a second‐order false belief task leads to earlier success (Sullivan et al. 1994), implying that task complexity (and not conceptual novelty) accounts for the developmental lag. However, growing evidence suggests that cognitive complexity alone cannot fully explain the developmental trajectory of advanced ToM. In a longitudinal study of children between ages 6–10, Osterhaus and Koeber (2021a) showed nonlinear development in mastering advanced ToM measures that involved an understanding of the recursive nature of mental states (i.e., higher‐order false belief tasks and the double bluff story from the Strange Stories; Happé 1994). Children showed a steep growth curve between the ages of 6–7, followed by plateaued performance until age 10. This suggests a more stepwise function (mastery of a particular cognitive capacity) as opposed to more linear increases in information processing. Using cross‐sectional methods, Osterhaus et al. (2016) found significant interrelations and systematic transitions from failure to success across various advanced ToM measures of differing complexity. Latent class analysis revealed distinct groups of children who either succeeded across multiple domains or failed consistently, indicating that mastery of advanced ToM likely reflects the acquisition of new conceptual competencies rather than incremental cognitive maturation alone.

Osterhaus and Koerber (2021b) further explored the relation between children's first‐order ToM (including measures of early false‐belief understanding) and advanced ToM performance in a sample of 5‐ to 8‐year‐old children. They found significant correlations between performance on the ToM scales and three distinct factors of advanced ToM: social reasoning, recognition of social norm transgressions, and reasoning about ambiguity. These correlations remained significant even when controlling for children's language skills and EF (inhibitory control). These data, however, are consistent with both interpretations. On the one hand, they showed that individual differences in advanced ToM were not fully explained by children's ToM at earlier ages. This suggests that mastery of advanced ToM measures involves children registering differences in conceptual requirements necessary for each measure. On the other hand, however, the correlation might indicate that children are extending and refining their understanding of others’ mental states more broadly (i.e., their “theories of mind,” cf. Lagattuta et al. 2016), more consistent with children's development being explained by high‐level conceptual continuity and better information processing skills as they encounter more complex social scenarios. This view is further supported by Osterhaus et al. (2022), who used longitudinal data and a scaling approach to show systematic transitions in performance across diverse ToM tasks, revealing both structured developmental progressions and moderately stable individual differences.

Taken together, existing findings point to both continuity and discontinuity in the development of ToM, and to a role of EF that is not yet theoretically resolved. What remains unclear is whether EF primarily supports the management of increasing task complexity across development, or whether it plays a more targeted role at specific transition points when children must reorganize their understanding of recursive mental states. To disentangle the mechanisms behind the refinement of children's ToM skills and to understand the progression from first‐order to advanced ToM, longitudinal investigations are necessary that include the same measures of first‐order and advanced ToM across time. Only by tracking the same children with both early and advanced ToM measures can researchers determine whether early ToM skills are a prerequisite for later social‐cognitive development—and whether such progression reflects conceptual restructuring or increased task‐processing capacity. Such a longitudinal study would also help isolate the relation between ToM and children's developing EF, particularly in terms of the directionality of the relations across different points of time.

### 1.2 The Role of EF in ToM Development

EF is central to the debate between the conceptual‐development and complexity‐only accounts because it can facilitate ToM performance in two theoretically distinct ways: by enabling children to express an existing understanding under task demands, and by scaffolding conceptual change at developmental transition points. Although executive function comprises multiple interrelated components—such as working memory, inhibitory control, and cognitive flexibility—that are theoretically relevant for belief reasoning (see e.g., Carlson and Moses 2001; Davis and Pratt 1995; Frye et al. 1998; Olson 1993), the present study focuses on overall executive capacity as a broad index of children's domain‐general control processes. Our goal is not to differentiate among specific EF components, but to examine whether general executive capacity shows the longitudinal patterns predicted by the two competing accounts.

Consistent with the view that EF help children express their existing conceptual understanding of belief, reducing the inhibitory demands on a false belief measure improves children's performance (e.g., Carlson et al. 1998; Hala and Russell 2001, see also Setoh et al. 2016). EF capacities also appear to moderate children's ability to develop the conceptual capacities needed to represent others’ false belief: Children with stronger inhibitory control capacities show greater improvement on false belief measures when given structured training about others’ mental states (Benson et al. 2013) and children with better ToM capacities require fewer inhibitory control resources to infer others’ false belief states.

However, the nature of this association varies with age. Longitudinal work showed that EF at age 3 predicts later first‐order ToM at ages 4–5, whereas early ToM does not predict later EF (Marcovitch et al. 2015; see also Carlson et al. 2004; Hughes and Ensor 2007, Schneider et al. 2005), supporting a unidirectional influence from EF to ToM in the preschool years. Longitudinal studies on older children, however, show different patterns. Devine et al. (2016) examined children at age 6 and age 10, testing them on EF, ToM and advanced ToM measures. Significant concurrent relations emerged between EF and ToM performance at both time points, but ToM at age 6 did not predict EF at age 10, nor did EF at age 6 predict advanced ToM at age 10. Although performance on first‐order ToM measures, like the false belief task, predicted later performance on advanced ToM measures, there was less of a role of early EF on later advanced ToM performance. A smaller‐scale longitudinal study between 9‐ and 10‐year‐olds (Lecce et al. 2017) found no concurrent relations between advanced ToM and EF, but some cross‐lagged associations were present between working memory at age 9 and advanced ToM at age 10 (see also Austin et al. 2014).

Although EF is consistently associated with performance on both first‐order and advanced ToM tasks, it remains unclear whether—and to what extent—EF contributes to the conceptual shift required to engage in recursive reasoning about mental states. Addressing this gap is essential for clarifying whether EF merely facilitates task performance or plays a more fundamental role in supporting the development of increasingly complex representational structures in ToM.

The present study was also designed to address this gap. Using a longitudinal design that begins in preschool and extends into middle childhood allows us to examine not only whether early ToM predicts advanced ToM performance, but also whether executive functions contribute to this transition—and if so, whether they do so by supporting task complexity, conceptual change, or both.

### 1.3 The Present Study: Tracking ToM and EF Development

The present study, which is part of a broader longitudinal study on children's cognitive development (see also Köksal et al. 2025), tracked ToM and EF at three time points: age 4, age 5.5, and age 7.5. At age 4, children were given a set of ToM measures from the theory of mind scales (Wellman and Liu 2004). Those same measures were administered at age 5.5, as well as a second‐order false belief measure (Sullivan et al. 1994). At age 7.5, children were administered the same second‐order false belief measure as at age 5.5, as well as two other advanced ToM measures. This way, we could track children's ToM capacities at each time point using developmentally adequate tasks. Children also received at each time point a set of EF measures, including a working memory battery, which was the same at all three time points; the other two measures were selected to represent age‐appropriate measures at each time point. Children were also administered measures of general cognition and language to control for broader cognitive and linguistic abilities.

Our primary goal was to evaluate two competing accounts of ToM development: the conceptual‐development hypothesis, which posits that progression from first‐order to advanced ToM involves a qualitative, conceptual shift, and the complexity‐only hypothesis, which argues that the same conceptual understanding underlies both types of tasks, with differences in performance driven by general cognitive demands.

These accounts differ not only in their interpretation of what changes in ToM development, but also in their predictions about developmental continuity. On a conceptual‐development view, early ToM provides a necessary foundation for advanced ToM, but the transition requires new conceptual insights. Thus, we expect ToM at Time 1 to predict later advanced ToM, but this continuity may break down between ToM at Times 2 and 3 if they fail to undergo the conceptual restructuring needed to master advanced ToM. Critically, EF should play a role in supporting this transition, specifically at key developmental junctures (e.g., from age 4 to 7.5), rather than uniformly across time points.

In contrast, the complexity‐only account assumes that first‐order and advanced ToM tasks share the same conceptual basis, with performance differences emerging from increasing task demands. This view predicts that early ToM and EF should consistently predict later advanced ToM in a linear fashion, and that EF should exert cross‐lagged effects on ToM at all stages of development, because advancing ToM performance reflects increasing information processing demands. On this view, advanced ToM tasks are expected to place particularly strong demands on executive processes involved in maintaining multiple representations and suppressing reality‐based responses. In contrast, the conceptual‐development account predicts that EF should be most predictive at developmental periods when children are transitioning to new forms of reasoning, with effects that are time specific rather than uniformly cross‐lagged.

To test these predictions, we used two complementary analytic approaches. First, cross‐lagged structural equation modeling (SEM) allowed us to examine the direction and consistency of longitudinal relations between EF and ToM. According to the complexity‐only view, EF should predict ToM at all time points. The conceptual‐development view, by contrast, predicts that EF will facilitate ToM primarily at specific points of conceptual transition (e.g., from age 4 to 7.5) but not uniformly across the developmental window. Because EF tasks necessarily change with age to remain developmentally appropriate, we treat EF at each wave as a broad index of core EF capacity while also emphasizing the conceptual relevance of inhibitory control and working memory demands for belief reasoning.

Second, we used latent class analysis (LCA) to identify subgroups of children following distinct ToM developmental trajectories. The complexity‐only account would predict more linear and continuous patterns across groups. However, the emergence of “partial achievers” (i.e., children who succeed at early ToM but not advanced ToM) would suggest a break in continuity and lend support to the conceptual‐development hypothesis. Such profiles would indicate that early ToM is not sufficient on its own, and that additional conceptual restructuring is required for successful advanced ToM performance.

Together, these approaches allow us to assess whether advanced ToM development reflects incremental increases in complexity or qualitatively new conceptual understanding, thereby clarifying how domain‐general and domain‐specific processes interact in shaping social‐cognitive growth.

## 2 Methods

### 2.1 Participants

The initial sample at Time 1 comprised two hundred three 4‐year‐olds (*M* = 48.00 months, *SD *= 1.53, range = 44–51; 90 girls, 113 boys). This baseline age range was chosen to capture children close to their fourth birthday, with most falling within a narrow window (*SD* = 1.53). This decision was informed by previous work using these tasks in both longitudinal and cross‐sectional studies, where children around age 4 demonstrated sufficient variability in ToM performance to allow meaningful longitudinal prediction. At Time 2, one hundred eighty of these children at age 5.5 years participated in the study (*M* = 64.97, *SD* = 1.45, range = 61–70; 82 girls, 98 boys). At Time 3, 158 of these children at age 7.5 years participated in the study (*M* = 88.93, *SD* = 1.52, range = 86–96; 73 girls, 86 boys). An additional 19 children were initially recruited. Of these, 17 children were excluded due to insufficient language skills assessed by a standardized measure (Grimm 2015); two additional children refused to take part in the study. An a priori power analysis was conducted using G*Power 3.1 (Faul et al. 2009) for a point‐biserial correlation, assuming a medium effect size (|ρ| = 0.30), *α* = 0.05, and power (1–*β*) = 0.95. This analysis indicated that a minimum sample size of 134 participants was required to detect significant effects. We recruited a larger sample in the initial cohort (*N* = 203) to allow for attrition over the longitudinal study and ensure sufficient power for more complex analyses, including structural equation modeling and latent class analysis. Children were recruited from predominantly White, middle‐class families in an urban area of Germany through local birth registries, and they did not have any diagnosed developmental disorders or hearing impairments. Ethics approval was granted by the ethics committee of the [blinded]. Parental consent and child assent were obtained for all participants. Parents were compensated for their travel expenses; children received a small gift at each time point for their participation.

Maternal and paternal education levels were measured at Time 1. Most parents held a university degree (74% of the mothers; 73% of the fathers), followed by a high‐school degree (12%; 14%), and a vocational degree (11%; 11%). An advanced degree was held by 1% and 1.5% of the mothers and fathers, respectively. Two families did not answer these questions.

### 2.2 Materials and Procedure

At each time point, children were administered a set of ToM measures, a set of EF measures, and at Times 1 and 3, control measures of general cognition. Other measures of causal and scientific reasoning and argumentation ability were also administered, but are described elsewhere (Authors 2025a, 2025b).

#### 2.2.1 Measures at Time 1 (Approximately 4 Years Old)

**ToM Measures**. We assessed children's ToM reasoning at Time 1 with three tasks taken from the German Theory of Mind Scales measure: knowledge access, content false belief, and explicit false belief (Kristen et al. 2006; Wellman and Liu 2004). These tasks were selected to capture different facets of children's early understanding of belief and its precursors. The knowledge access task assessed children's ability to understand that others may have access to different information than they do. Children were shown a toy dog figure inside a box and were subsequently questioned about whether a person who lacked visual access to the box know what is inside of the box. The content false belief task assesses children's understanding that others can hold false beliefs about the contents of an object. Children were shown a candy box (Smarties) containing an unexpected toy pig and asked to evaluate the belief of another individual who is unaware of the box's true contents. The explicit false belief task assesses children's ability to understand that others can hold false beliefs about the world. Children were presented with a story about a character who holds a belief that his gloves are in a wardrobe, despite them being in his backpack. The children were asked to predict where the character would search for his gloves. For each of these tasks, children were scored if they responded correctly or incorrectly.

**EF Measures**. The EF tasks were selected to represent the three core components of executive function (inhibition, working memory, and cognitive flexibility) as outlined in foundational models of EF development (e.g., Carlson 2005; Kloo and Sodian 2017). The design of the battery was guided by developmental considerations, with tasks chosen to be age‐appropriate while still tapping the relevant EF domain. Because EF tasks must vary across age to remain developmentally appropriate, the specific tasks differed across waves, with the exception of the Backwards Digit Span, which was administered at all three time points. Consistent with evidence that EF develops from a unitary structure in early childhood to a more differentiated two‐ or three‐factor structure in middle childhood, we treated EF at each wave as a composite index of core executive capacity. Principal component analyses (see Results section) supported this approach, as EF tasks at each time point loaded on a single component. Moreover, EF composites showed significant longitudinal continuity across waves, suggesting that the batteries captured a stable underlying executive construct rather than task‐specific variance.

At Time 1, Children's EF performance was assessed with three measures. First, children were given the Backwards Digit Span subtest of the HAWIVA‐III (Ricken et al. 2007). Children were presented with a series of digits and asked to repeat the digits in reverse order. Starting with a two‐digit sequence, the length of the digit sequence progressively increases until eight digits. Children received a score based on the total number of sequences they could accurately recall in reverse (scores ranged between 0 and 8). This measure was administered at all three time points. ICC was 0.99 for this measure.

Second, we used the Truck Loading procedure (originally described by Fagot and Gauvain (1997), but adapted here from Kloo and Sodian 2017) to assess children's planning. Children were presented with a toy mail truck and colored party invitations to deliver to similarly colored houses on a paper playboard. Children had to load the invitations on the truck in reverse order to distribute them in an efficient way. There were four difficulty levels; each level consisted of two trials. The lowest difficulty level required children to sort the mail of two houses, and one new house was added for each successive difficulty level, with the maximum difficulty level including five houses. Children received a score ranging from 0 to 4 based on the greatest difficulty level they reached. ICC was 0.96 for this measure.

Third, we used the Day/Night Stroop task (Gerstadt et al. 1994) to assess inhibitory control. Children were required to say “night” to a drawing of the sun and “day” to a drawing of the moon. Children received a score based on the sum of correct responses out of 16 test trials. ICC was 0.98 for this measure.

**Measures of General Cognition**. We administered two measures of general cognition at T1. First, children's language, memory, sentence comprehension, and morphological rule formation were assessed with three subtests of SETK 3–5 (Sprachentwicklungstest für Drei‐ bis Fünfjährige; Grimm 2015). Raw values from each subtest were transformed into standardized *T*‐values according to the age‐specific norms and the average of the three subtests was used as an index for children's verbal ability. ICCs for the three subtests ranged between 0.83 and 0.96 for these measures, indicating good to excellent interrater reliability.

Second, the Block Design subtest from the Hannover‐Wechsler Intelligence Test (HAWIVA‐III; Ricken et al. 2007) was administered as a proxy of nonverbal ability. Children were presented with a collection of red and white blocks and tasked with recreating specific designs and patterns using these blocks. The test measured their spatial perception, visual‐motor integration, and problem‐solving abilities. The subtest consisted of 23 items, each progressively increasing in complexity. For the initial six items, children were given two attempts (scoring 2 for the first try correct, 1 for the second try correct, and 0 for incorrect). Items 7–23 were scored with 2 for a correct design and 0 for an incorrect one (maximum score = 46). If a child made three consecutive errors, the task was terminated, and the child received a score of 0 for the remaining items. ICC was 0.98 for this measure.

#### 2.2.2 Measures at Time 2 (Approximately 5.5 Years Old)

**ToM Measures**. The same three first‐order ToM measures were administered at Time 2, along with a fourth measure of second‐order false belief understanding. This measure was the birthday puppy story (following Sullivan et al. 1994), and it was added to capture the onset of recursive belief reasoning. Children were told a story in which a mother misinforms her son about his birthday gift to surprise him, and the child discovers the real present unbeknownst to his mother. During a later conversation, the grandmother asks the mother what the child is getting as his birthday present and what he thinks he is getting. Children were asked about their mother's false belief about the child's belief. Like the first‐order ToM measures described above, children were scored as responding correctly or incorrectly.

**EF Measures**. In addition to the Backwards Digit Span task described above, at Time 2, children were given a version of the Dimensional Change Card Sort task (DCCS; Zelazo 2006) to assess cognitive flexibility. Children were presented with cards featuring drawings differing in two dimensions: color and shape. In the initial pre‐switch phase, they were asked to sort the cards based on the color dimension, and later, in the post‐switch phase, the sorting rule was changed to the shape dimension. Then, children were given a more challenging border phase, in which a new dimension was introduced; children were instructed to sort the cards based on the presence or absence of a border: sorting by color when there was a border on the cards and by shape when there was none. Children exhibited ceiling performance in the pre‐ and post‐switch phases; we used their scores from the 12 border version trials. ICC was 0.99 on this measure.

Children were also given a “Simon Says” task (originally used by Strommen (1973), adapted here from Kloo and Sodian 2017). Children were instructed to perform an action (e.g., touch your nose) only when the instructions were preceded by “Simon says.” They were particularly told not to perform the action when the instructions were not preceded by “Simon says” (non‐Simon trials). We scored the number of times children correctly inhibited their action on the 10 non‐Simon trials. ICC was 0.90 for this measure.

#### 2.2.3 Measures at Time 3 (Approximately 7.5 Years Old)

**ToM Measures**. In addition to the second order false belief task children were given at Time 2, children were also given a third‐order false belief task and a measure that assesses the understanding of nonliteral speech. These Time 3 measures were selected to capture more advanced, recursive forms of ToM. For the third‐order false belief measure, we adapted the “football trainer” story (Liddle and Nettle 2006; Osterhaus and Koerber 2021a). Children were told about a character named Niklas who wants to join the school's soccer team but thinks that the coach will choose his friend David over him for the team selection. The coach thinks that both David and Niklas are good players, and he is also aware of Niklas’ concerns. Children were asked to judge whether two given statements about the coach's belief were true or false: (1) “The coach thinks that Niklas knows that he wants him on the team” (The correct answer is “no”) and (2) “The coach thinks that Niklas does not know that he wants him on the team” (The correct answer is “yes”). The criteria for correctly demonstrating third‐order false‐belief reasoning involved answering both questions accurately, resulting in a score of 1. Children who answered at least one of the questions incorrectly received a score of 0.

Children's understanding of nonliteral speech was assessed with an adaptation of the “double bluff” story (Happé 1994; Osterhaus and Koerber 2021a). Children were learnt that a soldier was captured by his enemies. When they asked him about the location of the weapon arsenal, the soldier uses a double bluff to hide its true location: He tells them the weapon arsenal's true location as he knows they will disbelieve him. Children were asked why the soldier said this. They were told either that the soldier said this because he knows that the enemies will not believe him, or that the soldier said this because that is where the weapons are hidden. Children who chose the correct answer (the first one) were given a score of 1, otherwise they were given a score of 0. The reliability of the 3‐item scale was acceptable, with McDonald's ω = 0.47.

**EF Measures**. As in Time 1 and Time 2, children were given the Backwards Digit Span task. In addition, children were also given Fruit Stroop and verbal fluency procedures.

The Fruit Stroop task assessed inhibitory control (i.e., the ability to suppress a dominant response and select a subdominant response). As the final score, we computed a time‐based measure based on Roebers et al. (2011) approach. Interrater reliability was excellent (ICC = 0.98).

The verbal fluency task (Roebers et al. 2012) evaluates children's ability to recall information stored in their semantic memory. Children were instructed to name as many different members of a given category as possible within a limited time. Initially, they underwent a 10‐s training session where they named as many colors as they could. In the two test trials, children first named animals and then named foods within 60 s. The unique number of items each child named in each trial was counted. The total verbal fluency score was calculated by summing the word counts from the two test trials. Approximately 30% of the data was coded by a second rater, demonstrating excellent interrater reliability (ICC = 0.99).

### 2.3 Analytic Approach

Our analysis proceeds in four steps. First, we consider relations among the ToM measures at the three time points and the relations among the three batteries of EF tasks. Second, we consider relations between the ToM measures and the EF measures at each time point. Third, we consider relations between the first and second time points, followed by relations between the second and third time points; we also conducted structural equation modeling (SEM) to assess the interplay between ToM and EF across all three time points.

Finally, to complement these variable‐centered approaches, we conducted a person‐centered analysis using latent class analysis (LCA). This approach was used to identify subgroups of children who followed distinct developmental trajectories in ToM performance across the three time points. Whereas regression and SEM approaches examine average trends across the sample, LCA enables us to investigate heterogeneity in ToM development—specifically, to assess whether early proficiency in first‐order ToM reliably predicts later mastery of advanced ToM, or whether children follow divergent developmental pathways (e.g., consistent high performers vs. late bloomers vs. partial achievers). In other words, this analysis allows us to investigate whether conceptual gains in ToM occur gradually, uniformly, or in discrete developmental shifts for different groups of children.

All regression and factor analyses were done using SPSS29 and all SEM were done using STATA18. The latent class analysis was conducted in depmixS4 (Visser and Speekenbrink 2010).

## 3 Results

### 3.1 Summary of Batteries

**FIGURE 1:**
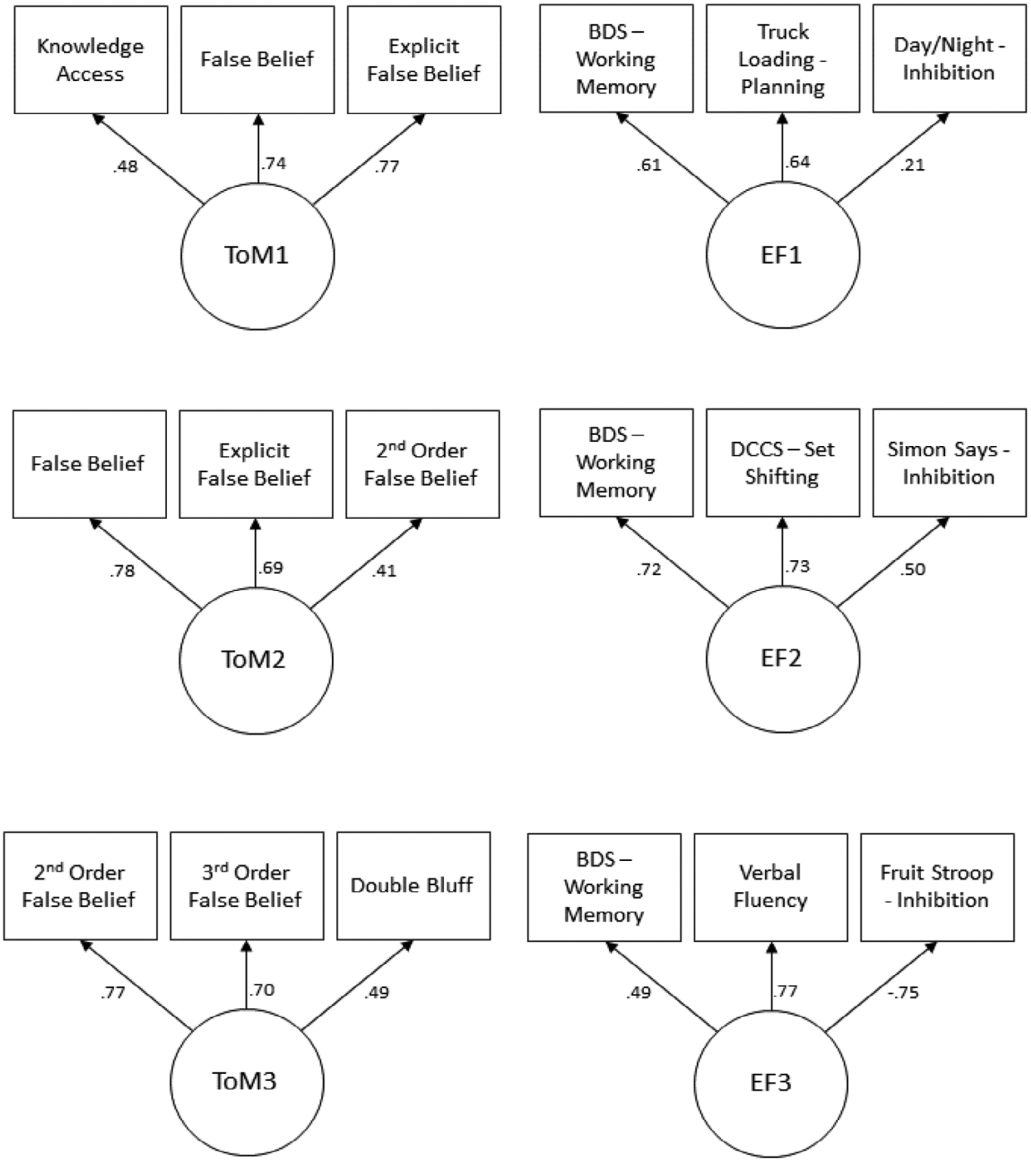
Results of principal components analysis for the ToM and EF Measures at each Time Point. The negative loading of the Fruit Stroop at Time 3 reflects that this measure was time‐based (lower scores indicate better inhibitory control).

We considered the relations among the ToM measures and among the EF measures administered at each time point via principal component analysis. For all but the ToM measures at time point 2, a single component was isolated. For the ToM measures at time point 2, the Bartlett's test of Sphericity was not significant, *p* = 0.10. This analysis was skewed by performance on the knowledge access measure being at near ceiling levels (94%). Although this task showed limited variance, the other ToM measures administered at Time 2—Content False Belief (*M* = 0.66, SD = 0.48), Explicit False Belief (*M* = 0.72, SD = 0.45), and Second‐Order False Belief (*M* = 0.35, SD = 0.48)—demonstrated substantial variability, mitigating concerns of a general ceiling effect. To avoid undue influence from the Knowledge Access task, we excluded it from the composite ToM score at this time point. When a PCA was conducted without the knowledge access measure, the Bartlett's test was significant, χ^2^(3) = 9.16, *p* = 0.03. Figure 1 shows the relations among each individual task and the component for that time point. Of note is that all KMO values for these analyses were between 0.50 and 0.70, suggesting that there is some uncertainty as to whether these variables each contribute equally to a common factor. As a result, and to account for missing data and cases in which the scoring systems were not equivalent, each measure was *z*‐scored,^1^ and those *z*‐scores were averaged to create a measure for analysis. This also allowed us to meet assumptions of normality for the analyses described below, as no measure met the criterion for a normal distribution on its own (all Shapiro–Wilk tests, *p* < 0.02). Averaging the *z*‐scores of each measure met the criteria for normalcy.

To describe these data, children passed 52% (SD = 32%), 58% (SD = 30%), and 72% (SD = 29%) of the ToM measures at Times 1, 2, and 3, respectively. At each time point, three measures of EF were administered. Summary scores on these measures are shown in Table 1.

**TABLE 1:** Mean performance on the ToM and EF measures across the three time points (standard deviations in parentheses).

	Score range	Time 1	Time 2	Time 3
ToM—Knowledge access	0–1	0.75 (0.44)	0.94 (0.23)	
ToM—Content false belief	0–1	0.38 (0.49)	0.66 (0.48)	
ToM—Explicit false belief	0–1	0.42 (0.49)	0.72 (0.45)	
ToM—Second‐order FB (Birthday)	0–1		0.35 (0.48)	0.65 (0.48)
ToM—Doube bluff	0–1			0.78 (0.41)
ToM—Third‐order FB	0–1			0.76 (0.43)
EF—Working memory (Backwards Digit Span)	0–8	1.79 (2.08)	4.20 (1.74)	6.28 (1.25)
EF—Planning (Truck Loading)	0–4	1.66 (1.15)		
EF—Inhibition (Day‐Night Stroop)	0–16	11.84 (4.00)		
EF—Cognitive flexibility (DCCS Boarder Condition)	0–12		7.17 (2.32)	
EF—Inhibition (Go‐No Go Simon Says)	0–10		10.43 (5.63)	
EF—Inhibition (Fruit Stroop)				29.94 (8.85)
Verbal fluency (Summary Score)				30.28 (7.51)

*Note*: Due to near‐ceiling performance on the knowledge access task at Time 2 (*M* = 0.94, SD = 0.23), this measure was excluded from the Time 2 composite ToM score and subsequent models to avoid distorting inter‐task correlations.

### 3.2 Relations Among ToM and EF

**ToM**. There was a significant relation between performance on the ToM measures at Times 1 and 2, *r*(171) = 0.31, *p *< 0.001. A similar relation held between Times 1 and 3, *r*(150) = 0.29, *p *< 0.001, but not between Times 2 and 3, *r*(156) = 0.11, *p *= 0.16. To investigate these relations more carefully, we constructed two hierarchical regression models in which ToM score at Time 2 or 3 was the dependent variable. In the first model, we considered children's age at Time 1, as well as their scores on the WPSSI and SETK 3–5 as measures of general intelligence at that time point. We then considered a second model in which the ToM score at Time 1 was added. When predicting ToM at Time 2, this second model explained a significant amount of additional variance, Δ*R* ^2 ^= 0.05, *F*(1,125) = 7.36, *p* = 0.008. When predicting ToM at Time 3, the additional variance explained by this second model was marginally significant, Δ*R* ^2 ^= 0.02, *F*(1,109) = 3.30, *p* = 0.07.^2^ This suggests that ToM performance at Time 1 has some predictive value at later time intervals.

**EF**. There were significant relations on the battery of EF measures between Times 1 and 2, *r*(176) = 0.39, *p *< 0.001, between Times 2 and 3, *r*(156) = 0.25, *p *= 0.001, and between Times 1 and 3, *r*(156) = 0.25, *p *= 0.002. To investigate these relations further, we built regression models looking at whether the later time point was predicted by age at the earlier time point, as well as measures of general cognition at that time point. First, we predicted EF at Time 2 with a hierarchical regression model with children's age, and scores on the WPSSI and SEKI at Time 1, and then added scores on the EF battery at Time 1. This second model explained a significant amount of additional variance, Δ*R* ^2 ^= 0.09, *F*(1,119) = 13.55, *p* < 0.001. We performed a similar regression predicting EF scores at time point 3 from EF scores at time point 1, which explained a significant amount of variance, Δ*R* ^2 ^= 0.05, *F*(1,105) = 5.69, *p* = 0.02. We also found that scores on the EF battery at time 2 explained a significant additional amount of variance in the EF scores at time point 3, Δ*R* ^2 ^= 0.03, *F*(1, 153) = 4.87, *p* = 0.03. Similar to the ToM measures, these analyses suggest that the EF measures have predictive value across the time intervals.

### 3.3 Concurrent Relations Between ToM and EF at Each Time Point

We next considered concurrent relations between the ToM and EF measures at each time point. This relation was significant at Time 1, *r*(186) = 0.32, *p* < 0.001, at Time 2, *r*(177) = 0.23, *p* = 0.002, and Time 3, *r*(156) = 0.25, *p* = 0.002. These relations were confirmed by hierarchical regression analysis. For each, the score on the ToM battery at that time point was the dependent variable. We first built a model with age, and performance on the measures of general cognition at each time point (WPPSI and SEKI at Time 1, WPPSI at Time 2, PPVT at Time 3), and then a second model with the EF score at that time point. At Time 1, that second model explained significant additional variance, Δ*R* ^2 ^= 0.04, *F*(1, 134) = 6.65, *p* = 0.01^3^. This was also true at Time 2, Δ*R* ^2 ^= 0.04, *F*(1, 173) = 7.16, *p* = 0.008, and Time 3, Δ*R* ^2 ^= 0.05, *F*(1, 151) = 8.09, *p* = 0.005.

To summarize, performance on the ToM measures related to each other across the time points, as did performance on the EF measures. Moreover, at each time point, ToM and EF measures are related to each other.

### 3.4 Cross‐Lagged Relations Between ToM and EF Across the Time Points

**FIGURE 2:**
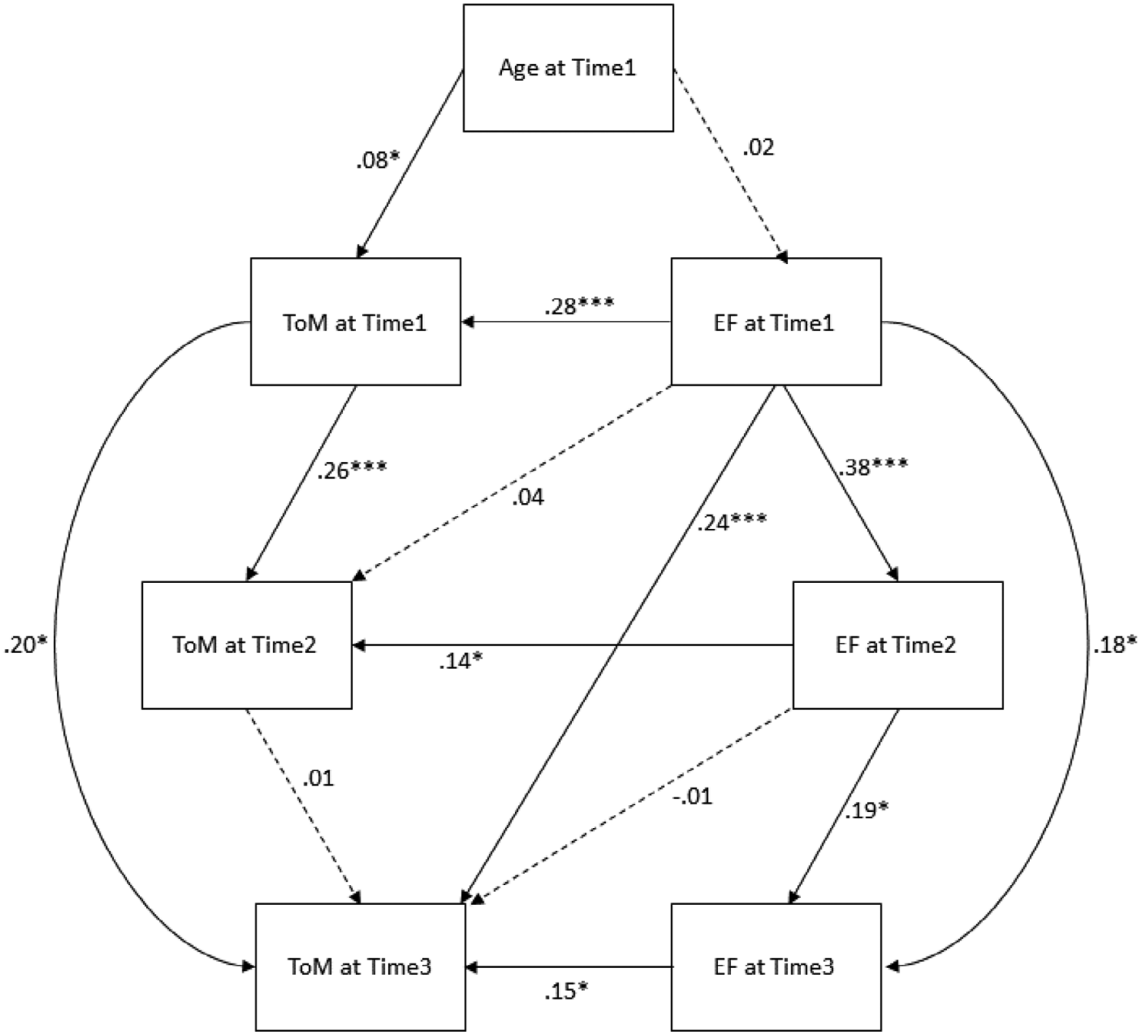
Structural equation model with parameter fits based on zero‐order correlation matrix for longitudinal variables between ToM and EF.

We next consider cascading (i.e., cross‐lagged) relations between ToM and EF across the time points we investigated via a Structural Equation Model. The guidelines for the model design were to examine the relations among the three ToM scores and three EF scores, as well as test whether there was a direct relation between EF at any time point and ToM at each and each subsequent time point. We posited relations based on the results of the regression analysis reported above. This model, with age at Time 1 as a predictor for both ToM and EF at Time 1 (Figure 2), showed a good fit for the data across all measures, χ^2^(7) = 10.36, *p* = 0.17, CFI = 0.97, TFI = 0.91, RMSEA = 0.049, 90% CI [.001, .11], *p_close_* = 0.45.

Children's ToM scores at Time 1 predicted their ToM score at Times 2 and 3, but no direct relation between ToM at Times 2 and 3 emerged. Similarly, EF scores at Time 1 predicted EF scores at Times 2 and 3, but in contrast to ToM, EF scores at Time 2 did predict EF scores at Time 3. At each time point, children's ToM and EF scores were related to one another. The concurrent association between EF and ToM was strongest at Time 1 (0.28), and substantially lower at Time 2 (0.14), and Time 3 (0.15) when the more complex measures of advanced ToM were employed. However, the relations from EF to ToM were not clearly longitudinal. EF scores at Time 1 did not predict ToM scores at Time 2, nor did EF scores at Time 2 predict ToM scores at Time 3. EF scores at Time 1, however, did predict ToM scores at Time 3.

This model was compared with several other models. The first was the same model, but with the connections between EF and ToM switched, so that ToM predicted EF instead of the other way around. This model did not provide as good a fit of the data nor did it meet the criteria for further analysis, χ^2^(7) = 20.53, *p* = 0.005, CFI = 0.88, TFI = 0.65, RMSEA = 0.098, 90% CI [0.05, 0.15], *p_close_* = 0.05. The BIC of this model was also higher than the BIC of the model shown in Figure 2 (3023.02 vs. 3012.84), so this model was not considered further.

The second was the same model shown in Figure 2, but with age replaced by a measure of general cognition at each time point; these measures of general cognition predicted the ToM and EF scores at that time point. This model also showed a good fit for the data—acceptable to be analyzed further—but not as good a fit as the model shown in Figure 2, χ^2^(15) = 33.72, *p* = 0.004, CFI = 0.89, TFI = 0.77, RMSEA = 0.078, 90% CI [0.043, 0.114], *p_close_* = 0.09. Of interest is that the measure of general cognition at Time 1 (the SEKI) predicted children's ToM score, *β* = 0.20, SE = 0.07, 95% CI [0.06, 0.33], *z* = 2.94, *p* = 0.003 and children's EF score, *β* = 0.32, SE = 0.07, 95% CI [0.19, 0.45], *z* = 4.93, *p* < 0.001. WPPSI scores at Time 2 predicted children's ToM scores, *β* = 0.35, SE = 0.07, 95% CI [0.22, 0.49], *z* = 5.05, *p* < 0.001, and EF scores at Time 2, *β* = 0.15, SE = 0.07, 95% CI [0.01, 0.29], *z* = 2.10, *p* = 0.03. Finally, PPVT scores at Time 3 predicted ToM scores at that time point, *β* = 0.21, SE = 0.08, 95% CI [0.05, 0.36], *z* = 2.61, *p* = 0.009, but not EF scores, *β* = 0.02, SE = 0.08, 95% CI [–0.13, 0.19], *z* = 0.31, *p* = 0.75. All the significant findings in Figure 2 were also present in this model.

### 3.5 Development of ToM and Advanced ToM: Person‐Centered Analysis

**FIGURE 3:**
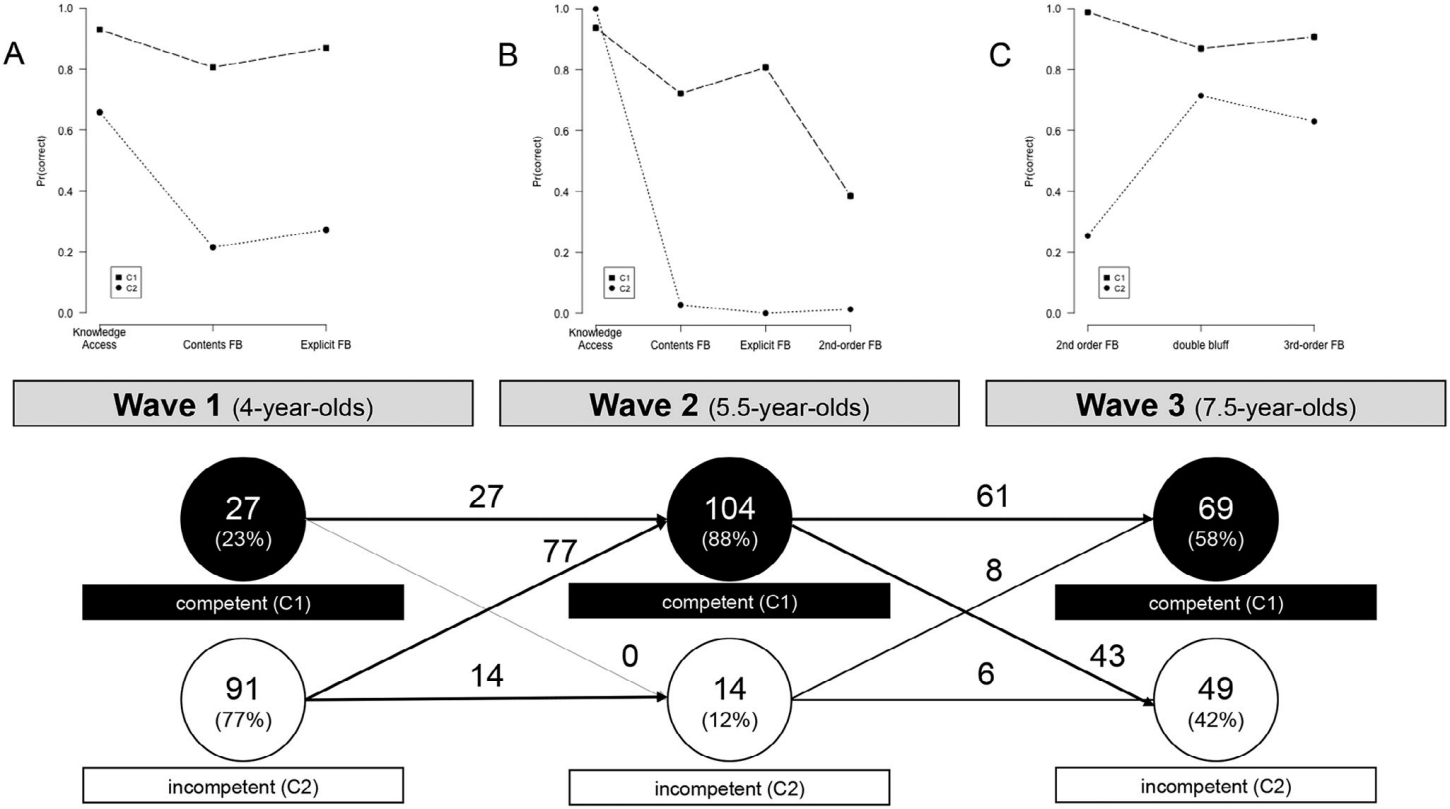
Latent class transition analysis of ToM proficiency across three time points (A–C). Children were classified as either competent (C1) or incompetent (C2) based on latent class analysis at each wave. Arrows indicate transitions between classes across waves. Numbers within boxes represent the number of children in each class at each wave; numbers on arrows indicate the number of children transitioning between classes.

To analyze individual response patterns within the ToM measures, we conducted a latent class analysis. The number of classes was determined using the Akaike Information Criterion (AIC). Across all waves, the optimal model identified was a two‐class model (see Table 2), delineating a group of proficient children from a group of less proficient ones (see Figure 3).

**TABLE 2:** Model comparisons latent class analysis.

	Log.Lik	Df	AIC	BIC
Time 1				
1‐class	−324.75	3	655.49	664.90
**2‐class**	−315.05	7	**644.10**	666.05
3‐class	−315.05	11	652.09	686.58
4‐class	−315.05	15	660.09	707.13
Time 2				
1‐class	−334.32	4	676.63	688.93
**2‐class**	−326.97	9	**671.94**	699.62
3‐class	−325.37	14	678.74	721.79
4‐class	−322.85	19	683.70	742.12
Time 3				
1‐class	−262.39	3	530.77	539.82
**2‐class**	−256.32	7	**526.64**	547.76
3‐class	−256.32	11	534.64	567.83
4‐class	−256.31	15	542.61	587.87

*Note*: The endorsed models are printed in bold.

Figure 3 presents the transition analysis. None of the children regressed to a lower state from Time 1 to 2, indicating that all children who attained mastery in false belief understanding at Time 1 maintained this proficiency at Time 2. However, nearly half of the children classified as proficient at Time 2 reverted to an incompetent state by Time 3, when measures of advanced ToM were used. This suggests that proficiency in first‐order ToM at Time 2 might not fully prepare children for tasks involving advanced ToM concepts. Most children identified in the competent group at Time 1 (*n* = 27) remained in the competent group at Time 3 (*n* = 21, 78%), indicating that early first‐order proficiency is predictive of early development of advanced ToM.

To investigate various developmental trajectories, we categorized children into four subgroups based on their ToM development as revealed by the latent class analysis: (1) *Consistently High Performers* were children who demonstrated competence in both ToM and advanced ToM across all time points (*n* = 26, 22%); (2) *Late Bloomers* were children who showed proficiency in ToM and advanced ToM at Times 2 and 3 but not at Time 1 (i.e., those who were in the competent profiles of the latent class analysis at time points 2 and 3, and in the incompetent group at time point 1; *n* = 36, 38%); (3) *Partial Achievers* were children who exhibited ToM competence at Time 2 but did not achieve proficiency in advanced ToM at Time 3 (*n* = 33, 34%); and (4) *Inconsistent Performers* were children with varied ToM competency across time points, such as competence at Time 1 but not Times 2 or 3 (*n* = 6, 6%).

Multinomial logistic regression was used to examine potential differences in developmental pathways concerning predictor variables. The model, which incorporated gender, language abilities (at Time 1), nonverbal reasoning, and inhibition (both at Time 3), yielded significance, χ^2^(9) = 19.19, *p* = 0.02, and explained 22% (Nagelkerke *R* ^2^) of the variance in the ToM developmental pathways. Significant effects were observed for language abilities at Time 1, χ^2^(3) = 9.24, *p* = 0.03, and inhibition at Time 3, χ^2^(3) = 8.35, *p *= 0.04, whereas nonverbal reasoning at Time 3 showed no significant effect, χ^2^(3) = 1.38, *p* = 0.71. Relative to the consistent high performers, both late bloomers and partial achievers exhibited significantly lower language abilities at Time 1 (OR = 0.91, 95% CI [0.83, 1.00], *p* = 0.05; OR = 0.88, 95% CI [0.80, 0.98], *p* = 0.02, respectively). Although language ability did not differ between the consistent high and inconsistent performers, the latter demonstrated significantly less inhibitory control at Time 3 (OR = 0.786, 95% CI [0.634, 0.974], *p* = 0.03). Finally, there was no significant association between the developmental pathways and children's gender, χ^2^(3) = 4.01, *p* = 0.26.

## 4 Discussion

This study investigated the development of ToM and EF across three time points in early childhood (ages 4, 5.5, and 7.5). We found significant relations regarding ToM performance between Times 1 and 2 and Times 1 and 3, but not between Times 2 and 3. EF, in contrast, showed consistent relations across all time points. Significant concurrent relations from EF to ToM were also found at each time point, with early EF at Time 1 also predicting advanced ToM at Time 3. Latent class analysis identified two distinct ToM proficiency groups per time point, and four developmental subgroups: consistently high performers, late bloomers, partial achievers, and inconsistent performers. Early language abilities and later inhibitory control were also significant predictors of ToM developmental pathways.

### 4.1 Conceptual Change or Complexity?

Our findings provide important insights into the ongoing debate about whether the transition from first‐order to advanced ToM reflects conceptual continuity with increasing task complexity (the complexity‐only hypothesis) or conceptual change requiring qualitatively new insights (the conceptual‐development hypothesis).

First, the cross‐lagged SEM results show that EF at age 4—but not at 5.5—predicted later ToM at age 7.5. This pattern is not consistent with the complexity‐only hypothesis, which would predict consistent EF‐to‐ToM effects across all time points. Instead, it suggests that EF plays a key role specifically at developmental transitions. At age 4, EF may serve two functions: helping children express their emerging understanding of false beliefs and supporting the conceptual restructuring required for more advanced recursive reasoning later in development. By age 5.5, most children have mastered first‐order ToM, and EF may no longer provide the same developmental leverage. Supporting this, Sodian et al. (2024) found that EF was related to children's performance on an explicit false belief task at age 4, but not on a lower‐demand version of the task. In our study, EF at age 4—but not 5.5—appears to facilitate the later acquisition of the representational capacities required for advanced ToM, highlighting its role in preparing children for conceptual change rather than merely supporting task performance.

Second, ToM at age 4 predicted ToM at age 7.5—even after accounting for EF and language—indicating that early ToM skills provide a foundation for later conceptual growth. This supports a view of conceptual continuity in development: early representational understanding plays a role in preparing children for later ToM success. This interpretation is consistent with findings from Osterhaus et al. (2022), who reported moderately stable individual differences and systematic transitions from failure to success across a broad range of first‐order and advanced ToM tasks, using Rasch modeling to demonstrate developmental sequencing. However, the lack of a predictive relation between ToM at age 5.5 and 7.5, coupled with the high rate of children moving from a “competent” profile at Time 2 to an “incompetent” profile at Time 3, suggests that continuity alone is insufficient. Rather, children may require an additional conceptual shift to master the recursive demands of advanced ToM tasks. This pattern aligns with findings from longitudinal studies (e.g., Osterhaus and Koerber 2023) showing nonlinear development in advanced ToM, with a steep increase around age 7, followed by a plateau phase, which is a finding that is consistent with a stepwise restructuring of conceptual understanding rather than gradual accumulation.

Third, the latent class analysis reinforces this interpretation. The emergence of “partial achievers” (i.e., children who succeed at first‐order ToM but not advanced ToM) would be unlikely under a purely complexity‐based account, which predicts that success on simpler tasks should translate linearly into success on more complex tasks. Instead, the presence of partial achievers strongly supports the conceptual‐development hypothesis, indicating a break in continuity as children move from first‐ to higher‐order reasoning.

Together, these findings support a hybrid view: there is high‐level conceptual continuity from early ToM to advanced ToM (as shown by Time 1 predicting Time 3), but also conceptual change at key developmental junctures (as shown by the EF effects and the partial achievers). EF and language appear to be especially important for supporting children during these transition periods, scaffolding both the expression of existing knowledge and the acquisition of new conceptual insights (San Juan and Astington 2012).

### 4.2 Developmental Pathways and Individual Differences

Previous research has established a significant association between ToM and EF in both preschool (e.g., Carlson and Moses 2001; Kloo et al. 2020) and in elementary school ages (e.g., Austin et al. 2014; Devine and Hughes 2016; Lecce et al. 2017; Wang et al. 2016). In preschoolers, findings indicate cross‐lagged relations between ToM and EF, with EF often predicting ToM (e.g., Marcovitch et al. 2015). In the present study, we did not find a predictive relation from EF at age 4 to ToM at age 5.5, even though both the ToM and EF tasks used across studies were similar; differences in assessment methods seem an unlikely cause for the divergence in findings.

An alternative explanation for the absence of a predictive relation is the performance pattern observed in our sample: at Time 1 (age 4), ToM performance was quite low, with the latent class analysis classifying only 23% as competent. This percentage increased dramatically to 88% at Time 2 (age 5.5 years). This high performance may have resulted in a ceiling effect and in restricted variance at Time 2, which may have made it difficult to detect cross‐lagged relations between individual differences between Times 1 and 2, masking potential predictive relations between early EF (at age 4) and later ToM development (particularly at age 5.5). This interpretation is in line with the strength of the concurrent association between ToM and EF at Time 2, which is substantially lower than the one at Time 1 (note: although it is similar in strength to the one at Time 3).

Although this account may partially explain the lack of cross‐lagged effects, we view it as complementary to, rather than in conflict with, our primary interpretation: that EF is most influential at developmental transition points involving conceptual change. In fact, a ceiling effect at Time 2 would suggest that many children had already mastered first‐order ToM by that age, reducing variability and limiting the need for EF to scaffold further growth. This aligns with the idea that EF matters most before conceptual restructuring (e.g., at Time 1, when children are just beginning to acquire false belief understanding) and again when new conceptual demands emerge (e.g., at Time 3, with the introduction of recursive reasoning in advanced ToM). Thus, the absence of predictive effects between Times 1 and 2 may reflect a temporary stabilization in development, rather than evidence against EF's role more broadly.

Findings regarding the relation between advanced ToM and EF are less consistent than the ones for first‐order ToM during the preschool years. Some studies report concurrent but not cross‐lagged relations, while others find stronger cross‐lagged predictions of ToM by EF (Austin et al. 2014; Lecce et al. 2017). Our findings align with the view that early EF predicts later ToM. The model in which there were hypothesized paths from ToM performance to EF performance at each time point was not a strong fit to the data. In our best fitting model, there was a significant cross‐lagged path from EF at age 4 to ToM at age 7.5, supporting the hypothesis that early EF is a precursor to later ToM development.

The latent class findings further clarified individual developmental trajectories. Nearly all children either advanced from the incompetent profile to the competent group between Times 1 and 2 or remained within the competent group. However, the transition from Times 2 to 3 was different, with many children's ToM regressing from a competent profile at Time 2 to an incompetent one at Time 3 (41% of the competent children regressed to the incompetent profile, 36% of the sample). Children who consistently performed well on ToM and advanced ToM tasks had stronger early language skills and better inhibitory control at age 7.5. In contrast, partial achievers and late bloomers showed lower language skills early on. These findings echo work showing that both EF and language are crucial in helping children shift from implicit to explicit reasoning about beliefs (Osterhaus et al. 2016; San Juan and Astington 2012).

### 4.3 Strengths, Limitations, and Future Directions

One notable strength of our study is the comprehensive assessment of EF through the inclusion of a diverse array of EF measures. By incorporating multiple tasks that evaluate different components of EF, such as the Day/Night Stroop task for inhibitory control, planning tasks, and working memory assessments, we ensured a robust and multifaceted assessment of this cognitive skill, which allowed us to capture the nuanced variations in EF development and provided a more reliable basis for examining the relation between EF and ToM.

At the same time, because EF tasks must be developmentally appropriate, the specific measures differed across waves, with the exception of the working memory task. Although principal component analyses supported treating EF at each time point as a unitary composite and the EF composites showed significant longitudinal continuity, we cannot fully rule out that task variation may have influenced effect sizes. Future studies using longitudinal designs with partially overlapping or latent‐variable EF models could further strengthen conclusions regarding developmental specificity.

Although our study employed a limited number of ToM tasks at both the earlier and later time points, this was intentional. First‐, second‐, and third‐order false belief, as well as double bluff, are all measures involving (higher‐order) misrepresentations or deception. By focusing on these aspects of advanced ToM, our studies did not examine the heterogeneity of advanced ToM measures, which encompasses many conceptually and methodologically different tasks (Osterhaus and Bosacki 2022). As these tasks do not assess the same underlying construct (Osterhaus et al. 2016) and also show different developmental patterns (Osterhaus and Koerber 2021a), narrowing the scope of advanced ToM measures to focus only on measures that involve the recursive nature of belief allowed us to address the relation between ToM—both first order and advanced—and EF in a more focused manner.

Another limitation of our study is that we designed the a priori power analysis around the parametric analyses (regressions) used to analyze longitudinal data, and not the more exploratory latent class analysis or SEM analyses that we performed. Future research should aim to employ larger and more diverse sample sizes. This would enhance the generalizability of the findings and allow for more sophisticated statistical analyses. Additionally, incorporating a wider range of ToM tasks, especially those that capture the different subcomponents and developmental trajectories of advanced ToM, would provide a more comprehensive understanding of how EF relates to different aspects of ToM development. Moreover, children in several cultures show different developmental progressions in the ToM scales as the German children tested here (Yu and Wellman 2024). Testing children in other cultures might reveal different roles of EF in ToM development.

## 5 Conclusion

This study highlights the interplay between EF and ToM from early to middle childhood. While EF was consistently associated with ToM performance at each time point, our longitudinal analyses revealed that early EF at age 4—but not later EF—predicted advanced ToM at age 7.5, suggesting that EF plays a key role during specific periods of conceptual change rather than exerting a uniform influence across development. We also found that early ToM at age 4 predicted later ToM at age 7.5, supporting the view that early representational understanding provides a foundation for later social‐cognitive reasoning. However, the emergence of “partial achievers” and the lack of predictive continuity from age 5.5 to 7.5 highlight that this continuity alone is insufficient, and that additional conceptual restructuring is required to master advanced ToM tasks. Together, these findings support a hybrid account of ToM development, in which both continuity and conceptual change play a role. Early language skills and later inhibitory control were key predictors of distinct developmental pathways, underscoring the importance of both domain‐general and domain‐specific capacities in shaping ToM trajectories. These insights emphasize the importance of nurturing both EF and language skills in early childhood to promote not only performance on basic ToM tasks, but to foster the deeper conceptual insights that underpin advanced forms of mental state reasoning.

## Policy on Using ChatGPT and Similar AI Tools

During the preparation of this work, the authors used Chat Generative Pre‐Trained Transformer (ChatGPT; OpenAI, San Francisco, CA, USA) to enhance readability and language. After using this tool, the authors reviewed and edited the content as needed and took full responsibility for the content of the publication.

## Conflicts of Interest

The authors declare no conflicts of interest.

## Data Availability

The data that support the findings of this study are available on request from the corresponding author.

## Endnotes

1 Because the Fruit Stroop measure used in Time 3 was inverted (lower scores are better), the *z*‐score was inverted as well for our scoring.

2 A difficulty with these models is that a majority of children (54) did not participate in the WPPSI at time point 1. When these analyses were rerun with only the SEKI as a control variable, we observed similar patterns of results, Δ*R* ^2 ^= 0.06, *F*(1, 161) = 10.59, *p* = 0.001 for time point 2 and Δ*R* ^2 ^= 0.02, *F*(1, 143) = 3.79, *p* = 0.053 for time point 3.

3 As noted above, many children did not participate in the WPPSI at time point 1, which reduced the sample size for this analysis. When this was removed, and only the SEKI was used as a control measure, the analysis presented above was also significant, Δ*R* ^2 ^= 0.04, *F*(1, 175) = 8.25, *p* = 0.005.
